# SurvMarker: an R package for identifying survival-associated molecular features using PCA-based weighted scores

**DOI:** 10.1186/s12859-026-06461-9

**Published:** 2026-05-08

**Authors:** Dona Hasini Gammune, Tongjun Gu

**Affiliations:** 1 Versiti Blood Research Institute, 8727 W Watertown Plank Rd, Milwaukee, WI 53226 USA; 2 Data Science Institute, Medical College of Wisconsin, Milwaukee, WI USA; 3 Department of Biostatistics, University of Florida, Gainesville, FL USA

**Keywords:** Feature Selection; Weighted Score; Survival Analysis; Principal Components; Dimension Reduction

## Abstract

**Background:**

Identification of prognostic molecular features from high-dimensional molecular data is central to biomarker discovery in cancer and other complex diseases. Principal component analysis (PCA) is widely used for dimensionality reduction in survival studies, yet selecting individual features from principal components (PCs) remains challenging and often relies on arbitrary thresholds. To address this limitation, we developed SurvMarker, an R package that prioritizes survival-associated molecular features using a PCA-based scoring framework.

**Results:**

SurvMarker applies PCA to normalized molecular data, jointly evaluates PCs using multivariable Cox proportional hazards models, and ranks features by aggregating absolute loadings across survival-associated PCs. Feature significance is assessed using an empirical null framework with false discovery rate control. In both synthetic global-null and permutation-based null simulations, SurvMarker showed comparative or better false positive control, particularly in small-n, large-p settings, compared with LASSO Cox, Elastic Net Cox, and Partial Least Squares Cox, while maintaining well-calibrated null *p*-value distributions. In the TCGA-LAML cohort, SurvMarker achieved the best predictive performance among these methods for gene expression data, with a C-index of 0.78 and an overall time-dependent AUC of 0.882 with similar applicability to miRNA expression data. Compared with sparse PCA-based and fixed per-PC threshold approaches, SurvMarker also achieved better predictive performance and yielded more compact, stable feature sets across different PC settings*.*

**Conclusions:**

SurvMarker provides a robust, interpretable, and reproducible framework for identifying survival-associated molecular features from high-dimensional data. By combining survival-guided PC selection, weighted feature aggregation across PCs, and empirical null-based inference, it improves false discovery control, stability, and biological relevance, and offers a practical tool for biomarker discovery across multiple omics data types.

**Supplementary Information:**

The online version contains supplementary material available at 10.1186/s12859-026-06461-9.

## Background

High-dimensional molecular profiling has become indispensable for biomarker discovery and prognostic modeling in cancer and other complex diseases. In survival analysis, the number of molecular features (e.g., genes or miRNAs) typically far exceeds the sample size, necessitating robust strategies for dimensionality reduction or feature selection to stabilize inference, control false discoveries, and preserve biologically meaningful signals.

Penalized regression approaches, including LASSO Cox (LASSO-Cox) [1] and Elastic Net Cox (ENC) [2], are widely used in this setting. LASSO induces sparsity through an $\ell_{1}$ penalty, yielding interpretable models, while Elastic Net combines $\ell_{1}$ and $\ell_{2}$ penalties to better accommodate correlated predictors [2, 3]. Partial Least Squares (PLS) Cox regression (PLS-R Cox) [4] offers an alternative by constructing latent components that maximize covariance with survival outcomes. Despite their effectiveness, these methods have important limitations. LASSO often selects a single representative from correlated feature groups, potentially discarding relevant signals [3, 5]. Elastic Net partially mitigates this issue but may still yield unstable or overly complex models [2]. PLS-based approaches rely on latent components that are not directly interpretable at the feature level and can dilute weak but biologically meaningful effects [6]. More broadly, these methods may be sensitive to noise and instability in high-dimensional, correlated settings.

Principal component analysis (PCA) provides a complementary framework by transforming high-dimensional data into orthogonal components that capture dominant patterns of variation [7]. PCA-based approaches are particularly attractive for survival analysis due to their ability to mitigate multicollinearity and reduce dimensionality without strong modeling assumptions. Extensions such as sparse PCA improve interpretability by enforcing sparsity in component loadings [8]; however, these methods do not explicitly incorporate outcome information and may overlook weak but distributed survival signals [9].

Supervised PCA partially addresses this limitation by incorporating outcome information through an initial univariate screening step, followed by PCA on a reduced feature set [9]. Although effective, the initial screening step does not explicitly account for correlation among molecular features, which may affect feature retention. Moreover, supervised PCA is often implemented using only a small number of components, potentially missing survival-associated signals distributed across multiple latent dimensions.

Despite the widespread use of PCA-based methods, there remains no principled and widely adopted framework for selecting molecular features from principal components (PCs). A common heuristic is to select a fixed number of top-loading features from each component [10], an approach also adopted in our earlier work [11], but this approach relies on arbitrary thresholds, treats components independently, and fails to capture features with moderate yet consistent contributions across multiple survival-relevant components.

To address these limitations, we developed SurvMarker**,** an R package that introduces a data-driven framework for PCA-based feature selection in survival analysis. Rather than selecting features from individual components, SurvMarker integrates loading information across all survival-significant PCs, explicitly linking feature importance to survival relevance. PCA is applied to normalized high-dimensional molecular data, and survival-associated PCs are identified using multivariable Cox proportional hazards models, allowing for the simultaneous evaluation of all components while optionally adjusting for clinical covariates. This multivariable framework accounts for interdependencies among PCs and ensures that the contribution of each component is assessed conditionally on the others, leading to more robust and reliable identification of survival-relevant latent structures. Feature importance is then quantified by aggregating loadings across these PCs, weighted by their explained variance, yielding a unified and interpretable score for each feature.

Statistical significance is assessed using an empirical null distribution constructed from PCs not significantly associated with survival, enabling robust estimation of *p*-values and false discovery rates. This framework captures features with distributed effects across multiple latent dimensions, addressing a key limitation of both regression-based and conventional PCA-based approaches.

To our knowledge, this loading-based feature scoring strategy has not been previously reported. By integrating dimensionality reduction, survival association, and feature-level inference within a single framework, SurvMarker offers an interpretable and statistically grounded approach for identifying prognostic biomarkers in high-dimensional settings. Although it was developed and evaluated primarily using transcriptomic data, the framework is readily extendable to other molecular data types, including proteomics, metabolomics, and DNA methylation.

## Methods

### Methodology

SurvMarker provides a unified framework for PCA-based feature selection in survival analysis. Given a normalized expression matrix $X\in {\mathbb{R}}^{n\times p}$, PCA is applied to obtain the top *K* orthogonal components, ${{PC}_{1},\dots , PC}_{K}$ with corresponding variance explained ${\lambda}_{1},\dots , {\lambda}_{K}$. All PCs are jointly evaluated using a multivariable Cox proportional hazards model:

$$h\left( {t|Z} \right) = h_{0} \left( t \right)\exp \left( {\mathop \sum \limits_{k = 1}^{K} \beta_{k} \,PC_{k} + \gamma^{{\mathrm{T}}} C} \right). $$

Here, *h(t∣ Z)* denotes the hazard function at time *t* given predictors *Z*, and ${h}_{0}(t)$ is the baseline hazard function. The full set of predictors is given by $\it Z=({\mathrm{P}\mathrm{C}}_{1},\dots,{\mathrm{P}\mathrm{C}}_{K},\mathrm{C})$, where $\it {\mathrm{P}\mathrm{C}}_{k}$ represents the *k*-th PC and ${\beta}_{k}$ is the corresponding regression coefficient quantifying its association with survival. The vector $\it \mathrm{C}$ denotes optional clinical covariates (e.g., age or sex), with *γ * representing their associated regression coefficients. Survival-associated PCs are identified based on the significance of ${\beta}_{k}$ (default: *p*-value $<\hspace{0.17em}0.05$).

Feature importance is quantified by aggregating loading contributions across all survival-associated PCs:

$$ S_{j} = \sum\limits_{k \in K} {w_{k} } \left| {l_{jk} } \right|, $$

Where ${S}_{j}$ denotes the aggregated importance score for feature *j*, ${w}_{k}$ reflects the relative contribution of the *k*-th PC, and ${l}_{jk}$ represents the loading of feature *j* on the *k*-th PC.

By default, weights are defined based on variance explained:

$${w}_{k}=\frac{{\lambda}_{k}}{\sum_{h\in K}{\lambda}_{h}}.$$

To incorporate outcome relevance, we additionally consider an alternative weighting scheme that integrates both variance explained and strength of association with survival:

$${w}_{k}= \frac{{\uplambda}_{k}[-\mathrm{log}\left({p}_{k}\right)]}{\sum_{h\in K}{\uplambda}_{h}[-\mathrm{log}\left({p}_{h}\right)]},$$

where ${p}_{k}$ denotes the significance level from the Cox model for PC *k*. This formulation allows components with stronger survival association to receive greater weight, even if their variance contribution is relatively small. Both weighting schemes are implemented in the SurvMarker package, with variance-based weighting used as the default.

Absolute loadings are used because PCA loading signs are directionally indeterminate and does not affect the magnitude of a feature’s contribution to a PC; rather, the magnitude reflects the strength of contribution [5, 7]. This variance-weighted aggregation prioritizes features that consistently contribute to survival-relevant latent structure across multiple components.

Statistical significance is assessed using an empirical null framework. For each iteration *b=1,… ,B*, an equal number of PCs—matching the count of survival-significant PCs—are randomly sampled equally with replacement from those not significantly associated with survival to construct null feature scores:

$${S}_{j}^{\left(b\right)}=\sum_{k\in {K}^{\left(b\right)}}{w}_{k}^{\left(b\right)}\mid {l}_{jk}\mid .$$

**Fig. 1:**
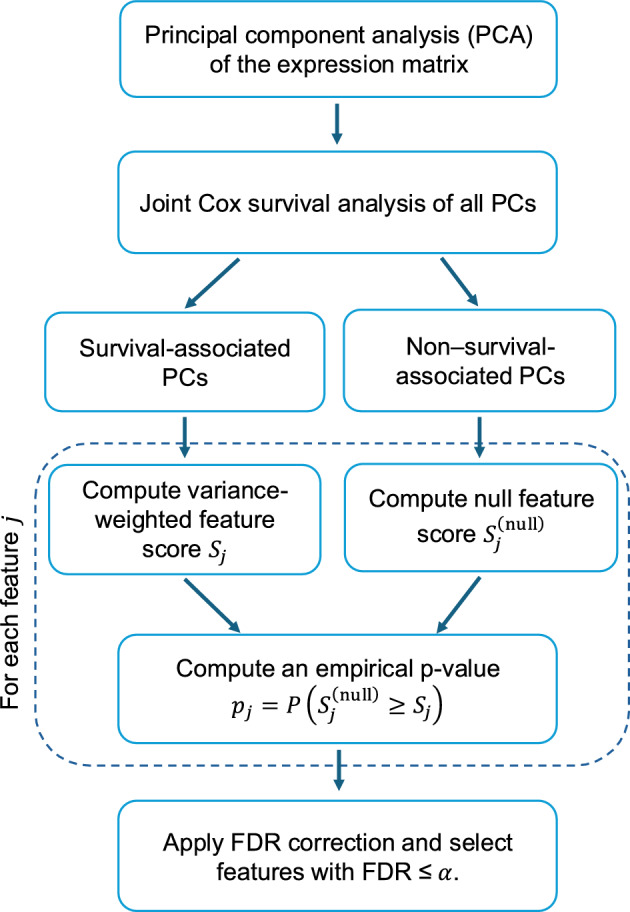
Workflow of the survival-guided PCA-based weighted feature scoring framework. Flowchart summarizing the SurvMarker analytical pipeline. The framework includes PCA of the normalized expression matrix, joint Cox proportional hazards modeling to identify survival-associated PCs, variance-weighted aggregation of feature loadings across selected PCs, empirical null construction, and FDR–controlled feature selection

Empirical *p*-values are computed as:

$$ p_{j} = \frac{1}{B}\mathop \sum \limits_{b = 1}^{B} I\left( {S_{j}^{\left( b \right)} \ge S_{j} } \right), $$

and adjusted using the Benjamini–Hochberg (BH) procedure to control the false discovery rate. The overall analytical procedure of SurvMarker is summarized in Fig. 1 and Additional File 1.

### Implementation

SurvMarker is implemented as an R package and distributed under the MIT License, enabling reproducible and flexible analysis of high-dimensional survival data. R was selected due to its widespread use in bioinformatics and its extensive ecosystem for statistical modeling and visualization. The package integrates seamlessly with existing genomic analysis workflows.

Core functionality leverages established R infrastructure. PCA is performed using prcomp [12], survival modeling is implemented via the survival package [13], and visualization utilities are based on widely adopted tools including ggplot2 [14] and VennDiagram [15]. This ensures computational robustness, transparency, and compatibility with standard analytical environments.

A typical analysis is performed using a single function call:

*pca_based_weighted_score(*

X = X, time = time, status = status, covar = covariates,

n_pcs = 50, cumvar_threshold = NULL, pc_cutoff = 0.05, feature_fdr_cutoff = 0.05, weight_type = c("variance", "outcome"), null_B = 500, scale_pca = TRUE, store_null = TRUE, seed = 1, …)

SurvMarker provides two complementary strategies for determining the number of PCs to be evaluated in the first step for identifying survival-associated PCs. Users may specify a fixed number of PCs through n_pcs, or alternatively select the minimum number of PCs required to explain a desired proportion of variance through cumvar_threshold, with max_pcs (default: 50 PCs) serving as a safety cap. Survival association is then evaluated jointly across all the PCs. Each component is assessed conditionally on the others. Survival-associated PCs are identified using a *p*-value threshold of 0.05 by default, with the pc_cutoff parameter allowing users to specify alternative thresholds. Feature selection is performed through aggregated scoring and empirical null calibration rather than fixed per-PC thresholds. In addition, users can specify the weighting scheme used for feature scoring, choosing between variance-based weighting (default) and an alternative outcome-informed weighting that incorporates both variance explained and strength of association with survival.

The output includes a ranked feature table with scores, empirical *p*-values, and adjusted *p*-values, along with PC-level summaries. Optional storage of null distributions and PCA objects enables reproducibility and supports downstream visualization.

**Fig. 2:**
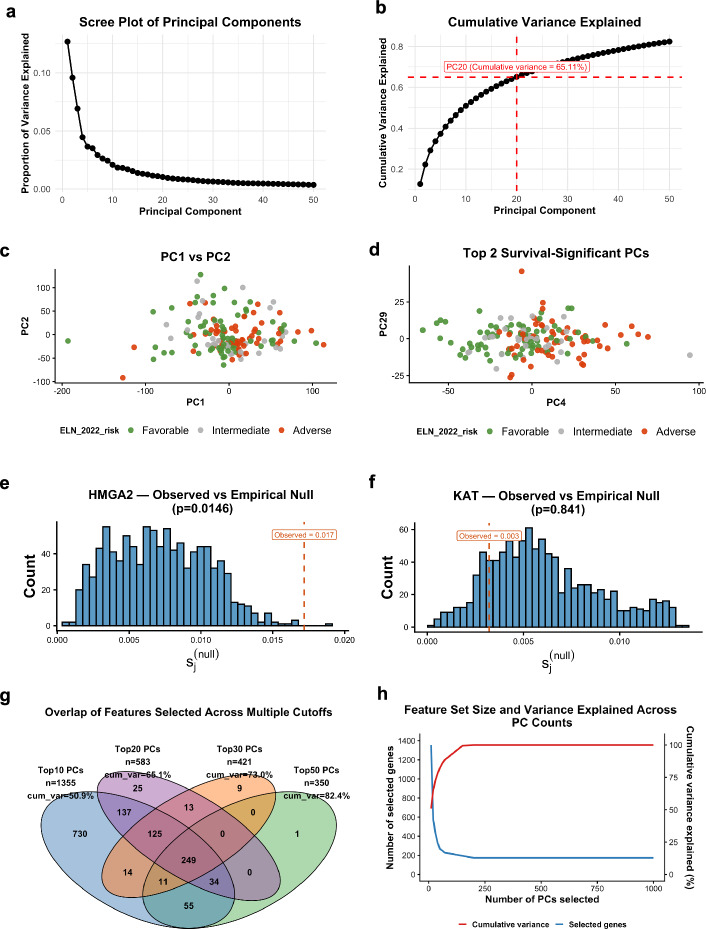
Implementation and visualization framework of survival-associated PCA-based feature scoring in SurvMarker. **a** Scree plot showing the proportion of variance explained by each PC. **b** Cumulative variance explained across PCs. **c** Scatter plot of PC1 versus PC2. **d** Scatter plot of the top two survival-associated PCs. **e**–**f** Empirical null distributions of the weighted feature score ${S}_{j}$ for a prognostically significant gene (*HMGA2*) and a non-significant gene (*KAT*), respectively. **g** Overlap of selected features across multiple PC thresholds; *n* denotes the number of selected features. **h** Trade-off between feature set size and cumulative variance explained as the number of included PCs increases

A typical usage example of SurvMarker is illustrated in Fig. 2, and the complete package reference manual and function documentation are provided in Additional File 2.

### Visualization, diagnostics, and sensitivity analysis

SurvMarker provides companion functions for diagnosing PCA structure and assessing feature stability. Functions such as plot_scree() and plot_cumvar() summarize variance explained across PCs, supporting transparent PC selection (Fig. 2a, b). Survival-associated latent structure can be visualized using plot_pc12() and plot_top2_survival_pcs(), which display component scores colored by clinical or molecular annotations (Fig. 2c, d).

Feature-level inference is supported by plot_null_vs_observed(), which compares observed feature scores to their empirical null distributions (Fig. 2e, f). Sensitivity analyses can be performed using run_survival_pca_multi_pc(), enabling evaluation across different PC configurations. Feature overlap and stability can be visualized using plot_venn() (Fig. 2g), while plot_feature_set_tradeoff() summarizes the relationship between variance explained and feature set size (Fig. 2h).

### Data preprocessing

Gene and miRNA expression profiles were obtained from the TCGA-LAML cohort (dbGaP accession: phs000178). Raw gene expression counts were normalized using the Upper Quartile (UQ) method [16], followed by ${log}_{2}$-transformation to stabilize variance and improve comparability across samples. To reduce background noise, genes with low expression (average expression < 1 count per million, CPM) [17] were excluded from downstream analysis. A similar preprocessing pipeline was applied to the miRNA expression data, and further details on preprocessing and quality control procedures are provided in our previous study [11].

### Simulation study design

We conducted two complementary simulation settings to evaluate the false positive behavior of the proposed method under realistic high-dimensional conditions. These settings were designed to assess robustness under both synthetic and data-driven null scenarios, where no true association exists between gene expression and survival outcomes.

#### Simulation setting 1: synthetic global null with structured correlation

In the first setting, data were simulated under a global null scenario in which no features are associated with survival. The gene expression matrix $X\in {\mathbb{R}}^{n\times p}$ was generated from a multivariate normal distribution:

$${X}_{i}\sim \mathcal{N}(\mu ,\Sigma ),$$

where gene-specific means and variances were estimated from the TCGA-LAML dataset. To mimic biologically realistic co-expression patterns, the covariance matrix *Σ * was constructed with a block-wise correlation structure, grouping features into blocks with shared pairwise correlation *ρ *.

To enforce the null condition, the linear predictor was set to zero for all individuals (${\eta}_{i}=0$), ensuring independence between gene expression and survival. Event times were generated from an exponential distribution with a constant baseline hazard, and independent censoring was applied to achieve realistic censoring rates. Survival times were calibrated to match the range observed in real data. Under this setting, the true set of survival-associated features is empty, and any selected feature represents a false positive.

#### Simulation setting 2: permutation-based null using real data

In the second setting, a data-driven null scenario was constructed using the TCGA-LAML gene expression matrix, preserving the intrinsic correlation structure among genes. Survival times and event indicators were randomly permuted across samples:

$$({T}_{i},{\delta}_{i})\to ({T}_{\pi (i)},{\delta}_{\pi (i)}),$$

where *π * is a random permutation. This procedure maintains the marginal distribution of survival outcomes while removing any association with gene expression.

This approach provides a realistic null framework in which biological structure is retained but all feature–outcome relationships are eliminated. As in the synthetic setting, the true set of associated features is empty.

### Comparative methods and evaluation metrics

We compared SurvMarker with widely used survival modeling approaches, including LASSO-Cox [1], ENC [2], and PLS-R Cox [4]. All methods were applied to each simulated dataset under identical conditions to ensure a fair comparison. For all models, tuning parameters—including the optimal number of components (*nt*) for PLS-R Cox—were selected using fivefold cross-validation. For ENC, the mixing parameter *α* was set to 0.5.

Each simulation was repeated 50 independent runs. Performance was mainly evaluated using two criteria: (i) the number of selected features, representing false positive discoveries under the null, and (ii) the concordance index (C-index) [18], which is expected to be approximately 0.5 in the absence of true predictive signal.

### Comparison of PCA-based dimension reduction methods

To evaluate the impact of different PCA formulations on survival-driven feature selection, we compared SurvMarker with several sparse PCA approaches, including classical sparse PCA (csPCA) [19], penalized matrix decomposition (PMA) [20], and elastic net–based sparse PCA (ensPCA) [21]. All methods were implemented using their default settings.

PCs were extracted from standardized gene expression data across multiple dimensionality settings (*K=10, 20, 30, 50*). Multivariable Cox models were fitted using these components, and survival-associated PCs were identified based on statistical significance.

Feature importance was computed by aggregating absolute loadings across significant PCs, with weights reflecting survival association. Statistical significance was assessed using the SurvMarker empirical null framework, in which PCs not statistically associated with survival were repeatedly resampled to generate null feature score distributions while preserving the correlation structure of the data.

This framework enables systematic evaluation of how different PCA formulations influence survival-associated component selection, feature identification, and robustness to spurious signals.

## Results

### Simulation study results

To assess false positive control under no true association, we conducted simulation studies under both global null and permuted null scenarios.

#### Global null scenario

**Fig. 3:**
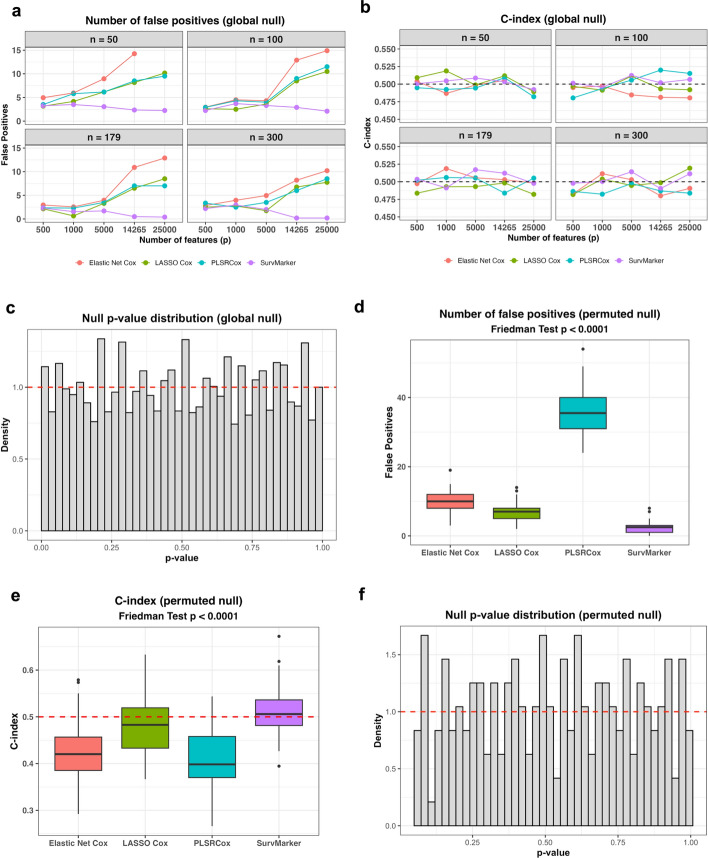
Performance of SurvMarker and other methods in simulation studies under global and permutation-based null scenarios. **a** Average number of selected features (false positives) under the global null. Results are averaged over 50 simulation iterations across varying sample sizes and feature dimensions; *n* in each panel denotes the sample size. **b** C-index under the global null. **c** Empirical p-value distribution under the global null. **d** Boxplots of the number of selected features under the permutation-based null setting (pairwise comparisons shown in Additional File 3c). **e** Boxplots of C-index under the permutation (pairwise comparisons shown in Additional File 3c). **f** Empirical p-value distribution under permutation

Under the global null scenario, where no association exists between gene expression and survival, SurvMarker demonstrated strong control of false positives compared to competing methods (Fig. 3). The number of selected features remained consistently low across simulation runs, indicating effective suppression of spurious discoveries. Compared to LASSO-Cox, ENC, and PLS-R Cox, SurvMarker achieved comparable feature selection in moderate settings, while demonstrating substantial improved control in high-dimensional scenarios with small sample sizes (*n≪ p*). In these challenging settings, it more effectively limited spurious feature selection, highlighting its robustness in low-*n*, high-*p* conditions (Fig. 3a). In addition, SurvMarker consistently achieved lower false positive rates across all simulation configurations, demonstrating improved control of type I error (Additional File 3a).

As expected under the null, all methods yielded C-index values close to 0.5 (Fig. 3b), indicating no predictive discrimination. Similarly, time-dependent AUC values remained approximately 0.5 across all evaluated time points (Additional File 3b), confirming the absence of survival signal and demonstrating that differences in feature selection are not driven by spurious predictive performance.

Importantly, the distribution of empirical *p*-values under the null was approximately uniform (Fig. 3c), indicating proper statistical calibration of the proposed framework. No evidence of *p*-value inflation or systematic deviation from uniformity was observed, supporting effective control of false discoveries.

Collectively, these results demonstrate that SurvMarker maintains robust control of false discoveries in challenging small-*n*, large-*p* settings, where conventional methods are prone to instability.

#### Permutation-based null scenario

Consistent results were observed under the permutation-based null scenario, where the intrinsic correlation structure of the data was preserved while feature–outcome associations were removed. Under this setting, SurvMarker continued to show strong control of false positives, selecting substantially fewer features than competing methods (Fig. 3d). In contrast, PLS-R Cox produced a notably high number of false positives, whereas LASSO-Cox and ENC also selected more features than SurvMarker, indicating greater susceptibility to spurious associations under permutation. These differences were statistically significant based on post hoc pairwise Wilcoxon signed-rank tests (*p*-value < 0.016; Additional File 3c), further supporting the superior performance of SurvMarker.

Despite these differences in feature selection behavior, predictive performance remained at chance level across all methods. C-index values were close to 0.5 (Fig. 3e), consistent with the absence of true signal. Post hoc pairwise Wilcoxon signed-rank tests showed that SurvMarker performed better than ENC and PLS-R Cox (*p*-value = 0.052 and 4.46 × 10^^−4^, respectively; Additional File 3d), while showing comparable performance to LASSO-Cox (*p*-value = 0.191).

Importantly, the distribution of empirical *p*-values under permutation was approximately uniform (Fig. 3f), indicating proper calibration of statistical significance. No evidence of *p*-value inflation or systematic deviation from uniformity was observed, supporting effective control of type I error in realistic data-driven null conditions.

Collectively, these results demonstrate that SurvMarker maintains robust false discovery control while avoiding spurious signal detection, even when the underlying correlation structure of the data is preserved.

### Real data analysis (TCGA-LAML)

#### Predictive performance

To evaluate the practical utility of the proposed framework in a real-world setting, we applied it to expression data from the TCGA-LAML cohort.

**Fig. 4:**
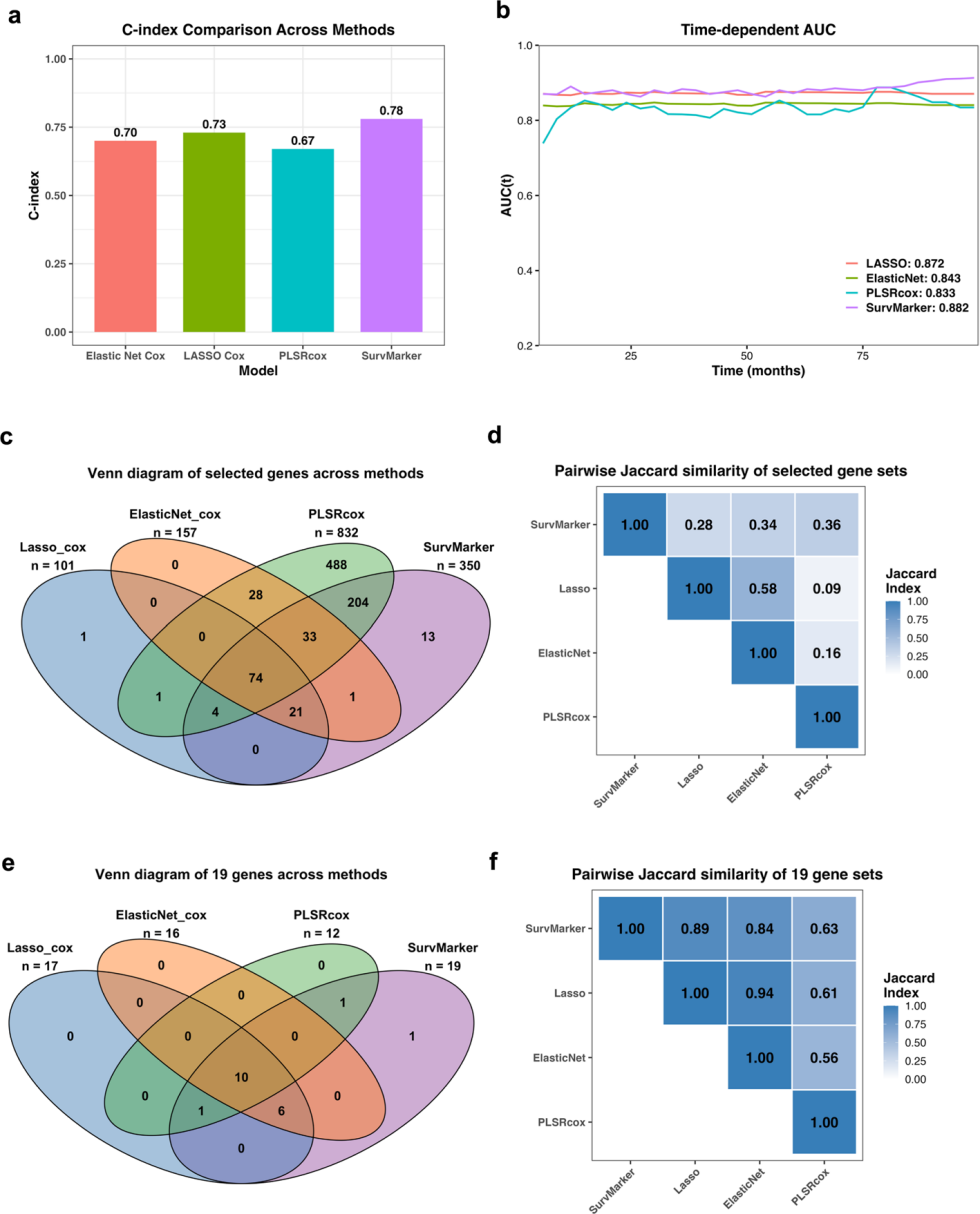
Performance of SurvMarker and other methods in real data analysis using the TCGA-LAML cohort. **a** C-index across methods. **b** Time-dependent AUC over follow-up time. **c** Overlap of selected gene sets across SurvMarker, LASSO Cox, ENC, and PLS-R Cox. n denotes the number of features selected. **d** Pairwise Jaccard similarity between selected gene sets. **e** Overlap of a subset of 19 selected genes across methods. **f** Pairwise Jaccard similarity for the 19-gene subset

SurvMarker achieved strong predictive performance on the gene expression data from TCGA-LAML dataset, outperforming competing methods in terms of C-index (Fig. 4a). Specifically, SurvMarker attained the highest C-index (0.78), compared with LASSO-Cox (0.73), ENC (0.70), and PLS-R Cox (0.67), indicating improved discriminative ability.

Time-dependent AUC analysis further demonstrated consistent and superior performance across multiple time points (Fig. 4b). SurvMarker achieved the highest overall AUC (0.882), followed by LASSO-Cox (0.872), ENC (0.843), and PLS-R Cox (0.833), indicating stable predictive accuracy over time.

Collectively, these results indicate that SurvMarker effectively captures survival-associated signal while maintaining competitive predictive performance relative to established methods.

#### Feature selection and biological validation

The number of selected features varied substantially across methods (Fig. 4c). PLS-R Cox selected the largest number of genes (n = 832), followed by SurvMarker (n = 350), ENC (n = 157), and LASSO-Cox (n = 101). This highlights fundamental differences in feature selection behavior across methods. LASSO-Cox selects a relatively small number of features due to the sparsity induced by the $\ell_{1}$ penalty, which shrinks many coefficients exactly to zero [1, 3]. While this promotes interpretability, it can be overly restrictive in high-dimensional genomic settings, where survival signal is often distributed across groups of correlated genes. As a result, LASSO-Cox may fail to capture complementary or jointly informative features [22].

Despite differences in feature set size, a shared subset of 74 genes was consistently identified across all methods, suggesting a core set of robust prognostic signals (Fig. 4c). Pairwise overlap, assessed using Jaccard similarity, showed the highest agreement between LASSO-Cox and ENC, reflecting their shared penalization framework (Fig. 4d). In contrast, SurvMarker exhibited moderate overlap with LASSO-Cox, ENC, and PLS-R Cox, indicating that it captures both shared signals and additional complementary genes beyond those identified by more restrictive methods.

To further assess biological relevance, we focused on a curated panel of 19 prognostic genes previously validated in our earlier study (Fig. 4e–f) [11]. SurvMarker successfully recovered all 19 genes, whereas LASSO-Cox and ENC identified 17 and 16 genes, respectively, and PLS-R Cox identified only 12 (Fig. 4e). Notably, 10 genes were consistently selected across all four methods (Additional File 4), representing a highly stable core signature. In addition, the only gene uniquely identified by SurvMarker, CXCL3, is a well-established AML-associated gene [23]. Together, these results demonstrate that SurvMarker can comprehensively recover known prognostic signals while remaining consistent with established markers. Pairwise Jaccard similarity within the 19-gene panel was substantially higher than that observed for the full feature set (Fig. 4f), indicating stronger agreement among methods within this set of previously validated prognostic markers. SurvMarker showed high similarity with LASSO-Cox (0.895) and ENC (0.842).

We further evaluated the impact of incorporating outcome-driven weighting into the feature scoring framework. The alternative weighting scheme, which integrates both variance explained and strength of association with survival, produced highly consistent results compared to the original variance-based weighting, with substantial overlap in selected features (Jaccard similarity approximately 0.88–1.00) (Additional File 5). This consistency was observed for both the full set of selected genes and the curated prognostic gene panel. These findings suggest that the initial filtering step—restricting to survival-associated PCs—already captures the most relevant components, resulting in minimal practical differences between weighting strategies.

To further assess statistical reliability, we examined the empirical null distribution constructed from PCs not significantly associated with survival. As shown in Additional File 6, *p*-values for PCs that are not statistically significant with survival were approximately uniformly distributed across different PC configurations (20, 50, 100, and 150 PCs), indicating appropriate calibration of the survival association testing procedure. This supports the validity of the multivariable Cox model and confirms that these PCs capture background variation unrelated to survival. Consequently, the empirical null framework provides a well-calibrated reference for feature-level inference. By sampling from these PCs to construct feature-level null score distributions, SurvMarker accounts for the underlying correlation structure of high-dimensional data, enabling robust control of false discoveries.

To assess the generalizability of the proposed framework, we applied SurvMarker to miRNA expression data. Consistent with the gene expression analysis, SurvMarker achieved predictive performance comparable to LASSO-Cox, ENC, and PLS-R Cox, while maintaining robust feature selection and well-calibrated statistical inference. Detailed results are provided in Additional File 7, demonstrating the applicability of the framework across different omics data types.

#### Comparison with PCA-based dimension reduction methods (TCGA-LAML)

**Fig. 5:**
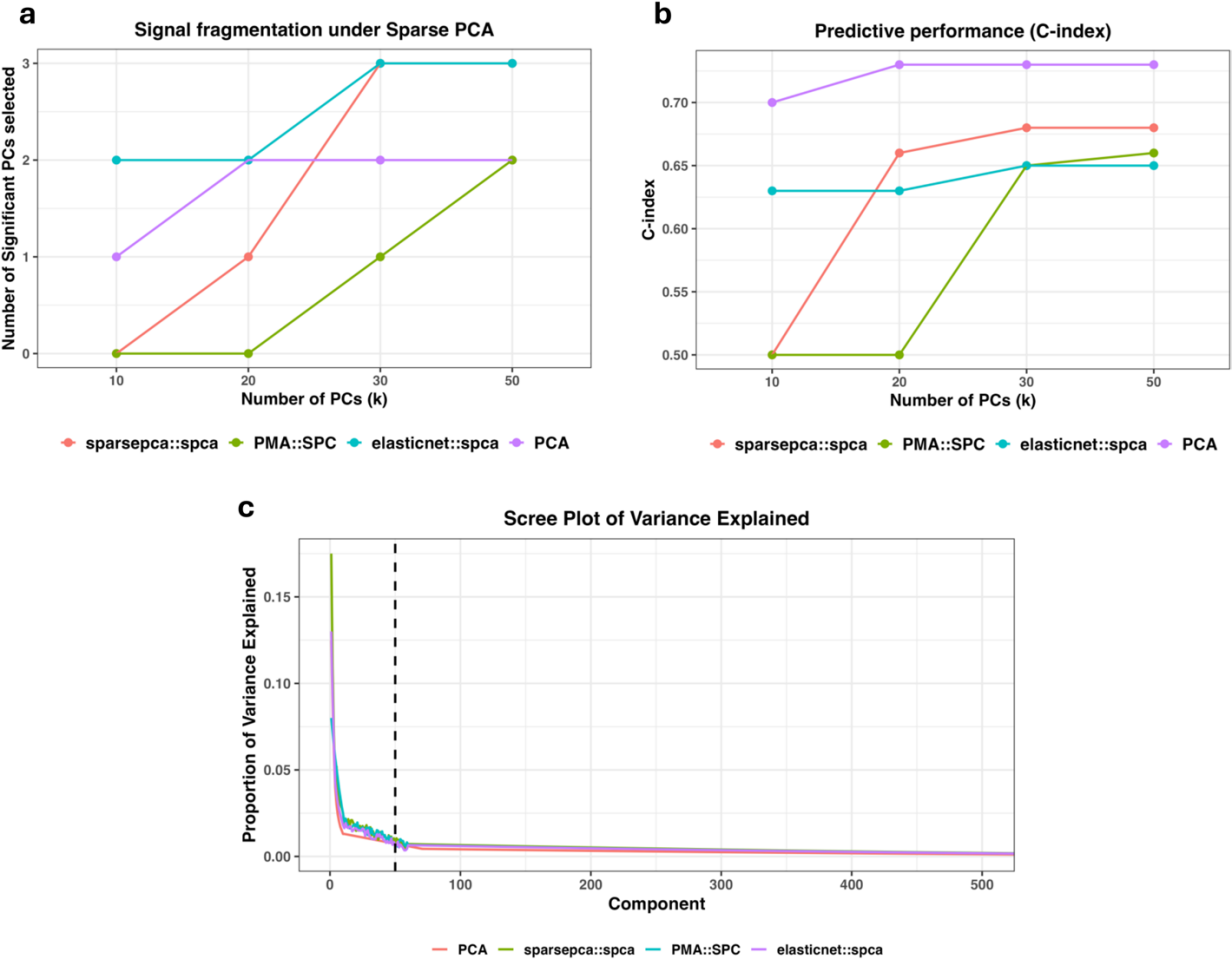
Comparison of PCA and sparse PCA–based dimension reduction methods. **a** Signal fragmentation under sparse PCA approaches **b** Predictive performance measured by the concordance index (C-index) across varying numbers of components (k). **c** Scree plot showing the proportion of variance explained by PCs. The dash line marks PC 50

We compared the proposed SurvMarker framework, which uses standard PCA for dimension reduction, with alternative PCA formulations, including csPCA, PMA, and ensPCA, all implemented using their default parameter settings (Fig. 5). Across different PC settings, sparse PCA and standard PCA produced broadly similar latent representations (Fig. 5a). However, sparsity constraints in sparse PCA set many loadings to zero, resulting in components constructed from subsets of features [8], whereas standard PCA captures contributions from all features and preserves a more global representation of the underlying latent structure [7].

This difference was reflected in predictive performance. Although sparse PCA methods showed comparable performance, SurvMarker consistently achieved higher C-index values, likely due to its ability to capture and aggregate survival signal distributed across correlated features (Fig. 5b).

These patterns are further supported by the variance structure captured by each method. As shown in the scree plot (Fig. 5c), standard PCA retains a relative larger proportion of variance in early components, whereas sparse PCA approaches distribute variance more diffusely, which may contribute to fragmentation of signal.

Taken together, these results suggest that, although sparse PCA methods offer improved interpretability, they may be less suited for settings where survival signal is distributed across correlated features, as commonly observed in genomic data. From a biological perspective, gene expression is governed by coordinated networks, and enforcing sparsity at the component level may disrupt these patterns [24, 25].

Importantly, the SurvMarker framework is not restricted to standard PCA and can incorporate alternative latent representations. However, in this study, PCA provided a stable and effective basis for capturing distributed signal, motivating its use within the proposed variance-weighted feature scoring framework.

#### Comparison with PC fixed-threshold selection

The fixed-threshold, per-PC selection strategy is widely used for feature selection based on PCA. To demonstrate the practical advantages of the proposed framework, we compared SurvMarker with this conventional fixed-threshold per-PC approach using gene and miRNA expression profiles from the TCGA-LAML cohort. Analyses were conducted on the full expression datasets and evaluated against established 19-gene and 16-miRNA prognostic panels. Unlike the fixed per-PC threshold strategy, which selects features independently within each component, SurvMarker integrates information across survival-associated PCs through a unified and statistically calibrated feature-scoring framework.

**Fig. 6:**
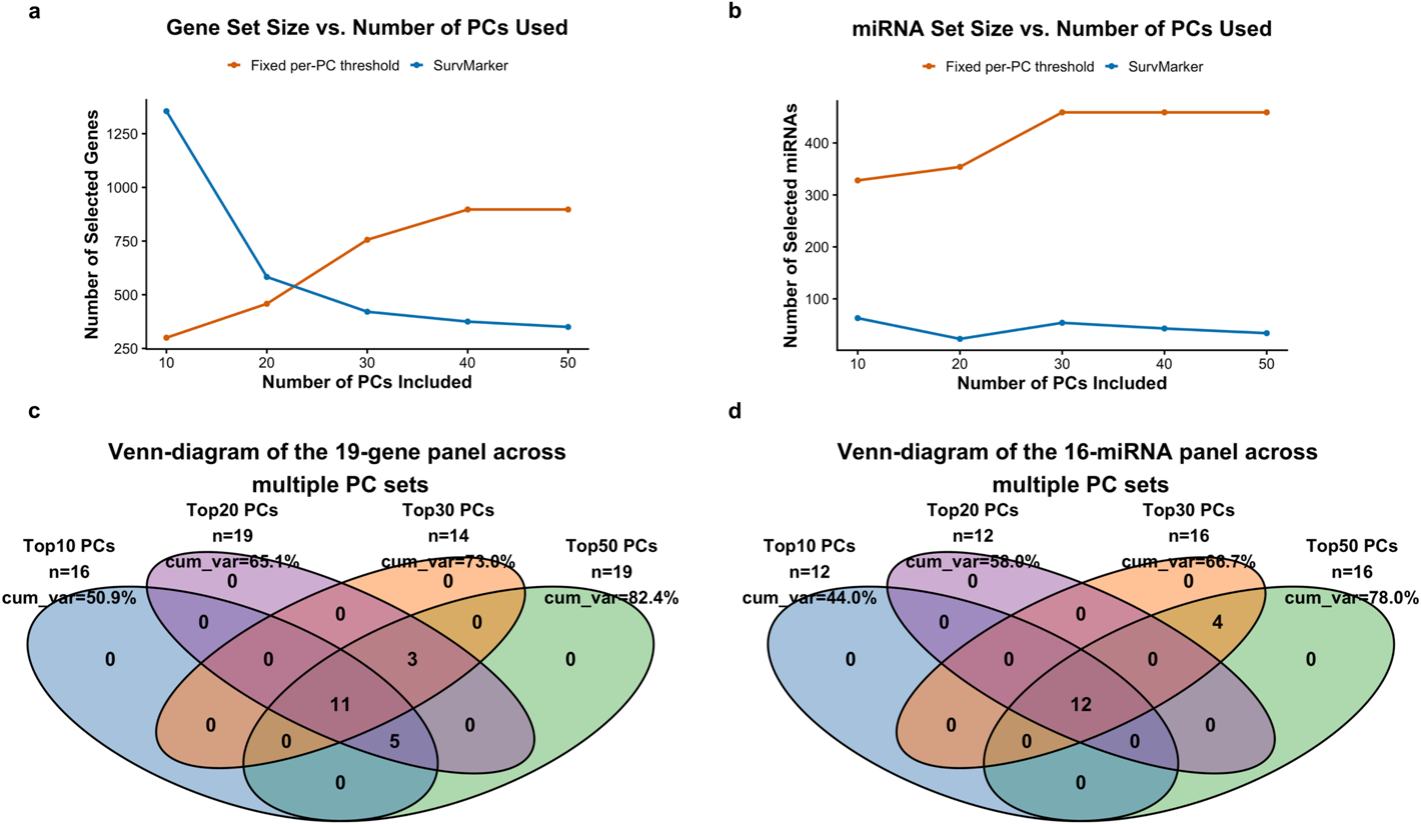
Comparative evaluation of SurvMarker and fixed per-PC threshold feature selection across varying numbers of PCs. **a**–**b** Number of selected genes (**a**) and miRNAs (**b**) as a function of the number of included PCs. The fixed per-PC loading threshold approach yields progressively larger feature sets as additional PCs are included, whereas SurvMarker maintains controlled and comparatively stable feature set sizes through variance-weighted aggregation of survival-associated PCs (consistent with Fig. 2g and Additional File 3). **c**–**d** Venn diagrams showing overlap of selected gene (**c**) and miRNA (**d**) panels derived from Top10, Top20, Top30, and Top50 PC configurations, indicating consistency of SurvMarker-selected features across varying PC settings

To enable a fair comparison, we evaluated candidate PC settings ranging from Top10–Top50. For each setting, we performed two parallel analyses: one using SurvMarker and the other using the PC fixed-threshold approach. As shown in Figs. 6a-b, the fixed-threshold approach leads to substantial inflation in feature set size as the number of PCs increases for both gene and miRNA data. This reflects the accumulation of features selected independently from each component, resulting in large and increasingly unwieldy feature sets. In contrast, SurvMarker consistently produces more compact and stable feature sets across Top10, Top20, Top30, and Top50 PC configurations. Notably, feature set sizes under SurvMarker remain stable or decrease as additional PCs are incorporated, indicating robustness to PC selection and reduced sensitivity to arbitrary truncation.

Venn diagrams (Fig. 6c-d) further highlight the stability and biological consistency of the selected prognostic panels. Across PC configurations, 11 of the 19 prognostic genes and 12 of the 16 prognostic miRNAs are consistently retained, defining a stable core signature. Additional features are introduced gradually as more PCs are considered, reflecting controlled refinement rather than abrupt, parameter-driven variability observed under the fixed-threshold approach.

Collectively, these results demonstrate that SurvMarker achieves a more principled balance between feature selection and model stability. By aggregating information across survival-associated PCs and calibrating feature importance using an empirical null framework, SurvMarker mitigates the accumulation of redundant or spurious features, reduces sensitivity to arbitrary parameter choices, and substantially improves reproducibility compared with fixed per-PC selection strategies.

## Discussion

We present SurvMarker as a variance-weighted feature scoring framework that leverages latent structure to identify survival-associated molecular features in high-dimensional data. Rather than positioning PCA as the primary contribution, our approach uses PCA as a stable and general basis for constructing feature-level scores, where importance is derived through aggregation across survival-relevant components. This shifts the focus from component-level modeling to feature-level inference, addressing a key gap in existing methodologies.

Compared with penalized regression methods such as LASSO-Cox [1] and ENC [2], which enforce sparsity directly on features, or PLS-based approaches [4] that rely on latent components, where the contribution of individual features is not directly interpretable, SurvMarker showed superior performance, particularly in the challenging *n≪ p* setting. A key advantage of SurvMarker is that it explicitly connects latent structure to interpretable feature scores. By aggregating contributions across multiple components, the method captures both strong and distributed signals, which are common in genomic data but often missed by approaches that rely on single-component selection or strict sparsity.

The proposed variance-weighted scoring strategy provides a principled mechanism for integrating information across latent dimensions, prioritizing stable and reproducible signals while mitigating noise by restricting to survival-associated components. We also explored an outcome-informed weighting scheme that incorporates both variance explained and strength of association with survival; however, this resulted in minimal differences in practice, suggesting that the variance weighted approach already captures the most relevant signal. Both weighting strategies are implemented in the package, providing flexibility for future applications, while the empirical null calibration ensures well-controlled feature-level inference by accounting for the underlying correlation structure.

More broadly, SurvMarker highlights the advantage of separating dimensionality reduction from feature selection. While the current implementation uses standard PCA, the framework is not restricted to a specific decomposition method and can incorporate alternative latent representations. However, our results suggest that methods enforcing sparsity at the component level may be less effective in biological settings where signals are inherently distributed, reinforcing the benefit of preserving full latent structure prior to feature scoring.

Several limitations of SurvMarker should be acknowledged. First, because the framework is based on PCA, an unsupervised linear dimensionality reduction method, the retained components primarily capture dominant variance patterns in the data, which may not always correspond to outcome-relevant biology. As a result, weak, nonlinear, or highly localized signals may be overlooked. Second, although our sensitivity analyses suggest that the method is relatively robust to the choice of candidate PCs, its performance may still be influenced by the selected component space, particularly in datasets with complex correlation structures. Third, the current evaluation focused primarily on transcriptomic data. Additional validation across independent cohorts, analytical platforms, and other omics data types will be important to further assess the generalizability of the framework.

Finally, the framework is inherently flexible and can be extended beyond survival outcomes. Although the current implementation is based on the Cox proportional hazards model, the same general strategy could be adapted to other outcome types by replacing the survival model with appropriate regression frameworks, such as logistic or linear models. Extending the framework in this direction is an important area for future work.

## Conclusions

SurvMarker introduces a variance-weighted feature scoring framework that enables robust and interpretable identification of survival-associated molecular features in high-dimensional data. By aggregating contributions across survival-relevant components and leveraging an empirical null model, the method achieves improved stability and control of false discoveries while maintaining strong predictive performance and preserving biologically meaningful signal. Its ability to recover validated prognostic markers while identifying additional candidate features highlights its practical relevance for biomarker discovery.

Implemented as an accessible R package, SurvMarker provides an end-to-end solution for survival-guided feature scoring, statistical inference, and visualization. The framework offers a flexible and generalizable approach for analyzing complex omics data and has the potential to facilitate reliable identification of prognostic markers in a wide range of biomedical applications.

### Availability and requirements

Project name: SurvMarker. Project home page: https://github.com/tjgu/SurvMarker. Operating system(s): Platform independent. Programming language: R (version ≥ 4.5.0). Other requirements: R packages: survival, ggplot2, VennDiagram (automatically installed via dependencies). License: MIT License. Any restrictions to use by non-academics: None.

## Supplementary Information

Supplementary Material1 (PDF)

Supplementary Material2 (PDF)

Supplementary Material3 (PDF)

Supplementary Material4 (PDF)

Supplementary Material5 (PDF)

Supplementary Material6 (PDF)

Supplementary Material7 (PDF)

## Data Availability

Gene expression and miRNA expression data were obtained from dbGaP under accession ID phs000178. All data generated and analyzed in this study are presented in the main figures and supplementary materials (Additional Files). The SurvMarker software is publicly available on GitHub at: https://github.com/tjgu/SurvMarker
