# Supplementary material for: SurvMarker: an R package for identifying survival-associated molecular features using PCA-based weighted scores
Source: BMC Bioinformatics. 2026 May 8;27:167. doi: 10.1186/s12859-026-06461-9 (PMC13449397; doi:10.1186/s12859-026-06461-9)
Supplement: Supplementary file 1 [file 12859_2026_6461_MOESM1_ESM.pdf]

Algorithm 1. PCA-based weighted feature scoring framework for survival analysis

Step 1: Survival-associated PC selection
Perform PCA on normalized expression matrix X and retain the top K PCs.
Fit a multivariable Cox proportional hazards model including all K PCs (optionally adjusting for covariates C):
$h(t \mid Z, C) = h_0(t) \exp\Big(\sum_{k=1}^{K} \beta_k z_{\cdot k} + \gamma^\top C\Big).$
Apply FDR correction across PC-level tests and retain survival associated PCs.
$\mathcal{K} = \{k : PC_k \text{ is survival-associated}\}.$

Step 2: Variance-weighted loading aggregation
for each $k \in \mathcal{K}$ **do**
Compute PC weight w_k (variance-based by default):
$w_k = \dfrac{\lambda_k}{\sum_{h \in \mathcal{K}} \lambda_h}.$
(Optional: outcome-informed weighting $w_k \propto \lambda_k[-\log(p_k)]$.) where λ_k is the eigenvalue of PC k.
end for
for each feature $j = 1, \ldots, p$ **do**
Compute observed feature score
$S_j = \sum_{k \in \mathcal{K}} |\ell_{jk}|\, w_k$, where ℓ_{jk} is the loading of feature j on PC k.
end for

Step 3: Empirical null construction and inference
Let $\mathcal{K}^c = \{1, \ldots, K\} \setminus \mathcal{K}$ denote the set of non–survival-associated PCs.
for each null iteration $b = 1, \ldots, B$ **do**
Randomly sample a subset $\mathcal{K}^{(b)} \subset \mathcal{K}^c$ with $|\mathcal{K}^{(b)}| = |\mathcal{K}|$.
for each $k \in \mathcal{K}^{(b)}$ **do**
Compute null weight $w_k^{(b)}$ using the same weighting scheme.
end for
for each feature $j = 1, \ldots, p$ **do**
Compute null feature score
$S_j^{(\text{null},b)} = \sum_{k \in \mathcal{K}^{(b)}} |\ell_{jk}|\, w_k^{(b)}.$
end for
end for
for each feature $j = 1, \ldots, p$ **do**
Compute empirical p-value
$p_j = \dfrac{1}{B} \sum_{b=1}^{B} \mathbb{I}\Big(S_j^{(\text{null},b)} \geq S_j\Big).$
end for
Apply FDR correction to $\{p_j\}$.
Select features with FDR-adjusted p-value $< \alpha$.
