# Supplementary material for: SurvMarker: an R package for identifying survival-associated molecular features using PCA-based weighted scores
Source: BMC Bioinformatics. 2026 May 8;27:167. doi: 10.1186/s12859-026-06461-9 (PMC13449397; doi:10.1186/s12859-026-06461-9)
Supplement: Supplementary file 2 [file 12859_2026_6461_MOESM2_ESM.pdf]

SurvMarker Reference Manual

SurvMarker: An R Package for Identifying Survival-Associated Molecular Features Using PCA-Based Weighted Scores

Description

SurvMarker implements a PCA-based weighted feature-scoring framework for survival analysis that identifies prognostically relevant molecular features by aggregating information across survival-associated principal components and assessing feature significance using an empirical null distribution.

Dependencies

`survival`, `ggplot2`, `VennDiagram`

Contents

`pca_based_weighted_score()` 1
`plot_scree()` 4
`plot_cumvar()` 4
`plot_pc12()` 5
`plot_top2_survival_pcs()` 5
`plot_null_vs_observed()` 6
`run_survival_pca_multi_pc()` 7
`plot_venn()` 7
`plot_feature_set_tradeoff()` 7

1. PCA-based Feature Scoring

`pca_based_weighted_score()`

Purpose: Performs survival-guided PCA to compute weighted feature scores, construct feature-specific empirical null distributions, and identify prognostically significant features. Feature scores aggregate consistent contributions across multiple survival-associated principal components, avoiding arbitrary per-component thresholds. Empirical *p*-values quantify score extremeness relative to null distributions derived from PCs with no survival association.

Usage

```
pca_based_weighted_score(
  X,
  time,
  status,
  covar = NULL,
  n_pcs = 50,
  cumvar_threshold = NULL,
  max_pcs = 50,
  pc_cutoff = 0.05,
  feature_fdr_cutoff = 0.05,
  null_B = 500,
  weight_type = c("variance", "outcome"),
  seed = 1,
  scale_pca = TRUE,
```

```
  store_null = TRUE,
  verbose = TRUE
)
```

Arguments

`X`	Numeric matrix or data frame of dimension *n_samples* × *p_features*. Rows correspond to samples and columns to molecular features (e.g., genes, miRNAs, proteins).
`time`	Numeric vector of survival times, in the same order as the rows of X.
`status`	Numeric or logical event indicator (1 = event, 0 = censored).
`covar`	Optional data frame of clinical covariates to include in Cox regression models.
`n_pcs`	Integer specifying the number of principal components to retain. Ignored if `cumvar_threshold` is provided.
`cumvar_threshold`	Numeric value in (0,1]. Selects the minimum number of PCs required to reach this cumulative variance threshold.
`max_pcs`	Hard upper bound on the number of PCs used (safety cap).
`pc_cutoff`	*p*-value cutoff for selecting survival-associated PCs.
`feature_fdr_cutoff`	False discovery rate cutoff for selecting prognostic features.
`null_B`	Number of empirical null resamples used for feature-level inference.
`weight_type`	Character string specifying the PC weighting scheme. Options are `"variance"` (default), which weights PCs by variance explained, and `"outcome"`, which incorporates both variance explained and strength of association with survival.
`seed`	Random seed for reproducibility.
`scale_pca`	Logical; whether to scale features prior to PCA.
`store_null`	Logical; whether to store the full empirical null score matrix.
`verbose`	Logical; whether to print progress messages.

Value

A list containing:

`feature_table`	Data frame containing feature loadings on survival-associated PCs, aggregated score (S_j), empirical p-values, and false discovery rates (FDR).
`pc_table`	PCA summary table including eigenvalues, proportion and cumulative variance explained, Cox regression coefficients, and adjusted p-values for each PC.
`pc_scores`	Sample-level principal component coordinates used for downstream visualization and clustering.
`null_scores`	Empirical null score matrix (features × `null_B`) returned when `store_null = TRUE`.
`selected_features`	Character vector of prognostically significant features passing feature-level FDR control.

Details

Let $\mathbf{X} \in \mathbb{R}^{n \times p}$ denote the normalized expression matrix with n samples and p features. PCA is applied to $\mathbf{X}$ (optionally after scaling), yielding principal component scores

$$\mathbf{Z} = \mathbf{XV},$$

where $\mathbf{V} = (v_{jk})$ contains the PC loading vectors and Z_{ik} denotes the score of sample i on PC k.

Identification of survival-associated principal components

For each retained principal component k, its association with survival is evaluated using a Cox proportional hazards model

$$h_i(t \mid Z_{ik}, \mathbf{c}_i) = h_0(t) \exp\left(\beta_k Z_{ik} + \boldsymbol{\gamma}^\top \mathbf{c}_i\right),$$

where Z_{ik} denotes the score of individual i on principal component k, $\mathbf{c}_i$ represents optional clinical covariates (e.g., age, sex, ELN-2022 risk group), β_k is the PC-specific log hazard ratio, and $h_0(t)$ is the baseline hazard function. *p*-values are adjusted for multiple testing across PCs using the Benjamini–Hochberg procedure, and PCs with adjusted *p*-values less than `pc_cutoff` are declared survival-associated.

Feature score aggregation

For each feature j, a weighted feature score S_j is computed by aggregating its loadings across all survival-associated PCs:

$$S_j = \sum_{k \in \mathcal{K}} w_k \; \ell_{jk},$$

where $\mathcal{K}$ denotes the set of survival-associated PCs, $\ell_{jk} = \mid v_{jk} \mid$, and

$$w_k = \frac{\lambda_k}{\sum_{h \in \mathcal{K}} \lambda_h}$$

is a variance-based weight proportional to the eigenvalue λ_k (variance explained) of PC k.

To incorporate survival outcome relevance, we also consider an alternative weighting scheme that integrates both variance explained and strength of association with survival:

$$w_k = \frac{\lambda_k \left[-\log\left(p_k\right)\right]}{\sum_{h \in \mathcal{K}} \lambda_h \; \left[-\log\left(p_h\right)\right]},$$

where $p_k$denotes the significance level of PC $k$from the Cox model.

The resulting score S_j reflects the overall contribution of feature j to survival-relevant latent structure.

Empirical null distribution and inference

Statistical significance of S_j is evaluated using an empirical null distribution constructed by repeatedly sampling PCs that are not associated with survival. For each null iteration $b = 1, \ldots, B$, a null score

$$S_j^{(b)} = \sum_{k \in \mathcal{K}^{(b)}} w_k^{(b)} \ \ell_{jk}^{(b)}$$

is computed using the same aggregation scheme, where $\mathcal{K}^{(b)}$ is a randomly selected set of non–survival-associated PCs of the same size as $\mathcal{K}$.

The empirical p-value is defined as

$$p_j = \frac{\sum_{b=1}^{B} \mathbb{I}\left(S_j^{(b)} \geq S_j \right)}{B},$$

and feature-level false discovery rates are controlled using the Benjamini–Hochberg procedure at level `feature_fdr_cutoff`.

2. Visualization Functions

2.1 PCA Variance Diagnostics

`plot_scree()`

Description

Plots the proportion of variance explained by each PC, with optional annotation of a selected PC cutoff.

Usage

```
plot_scree(
  res,
  n_pcs = 50,
  show_threshold = FALSE,
  threshold_pc = NULL,
  ...)
```

Arguments

`res`	Result object returned by `survival_based_loading_score()`.
`n_pcs`	Number of PCs to display.
`show_threshold`	Logical; draw a vertical line at `threshold_pc`.
`threshold_pc`	PC index for annotation.

Value

A `ggplot` object.

`plot_cumvar()`

Description

Plots cumulative variance explained across PCs with optional threshold annotation.

Usage

```
plot_cumvar(
  res,
  n_pcs = 30,
  show_threshold = FALSE,
  cum_threshold = 0.5,
  ...
)
```

Details

Facilitates principled selection of PC dimensionality.

2.2 PCA Scatter Plots

`plot_pc12()`

Description

Creates a scatter plot of two selected principal components with optional coloring and shaping by metadata.

Usage

```
plot_pc12(
  res,
  meta = NULL,
  id_col = "PATIENT_ID",
  pc_x = 1,
  pc_y = 2,
  color_by = NULL,
  shape_by = NULL,
  point_size = 3,
  ...
)
```

Arguments

`res`	Result object returned by `pca_based_weighted_score()`.
`meta`	Optional metadata data frame used for sample annotation.
`id_col`	Column name in meta identifying sample IDs.
`pc_x`, `pc_y`	Integer indices of the principal components plotted on the x- and y-axes.
`color_by`	Metadata column used to map points to colors.
`shape_by`	Metadata column used to map points to shapes.
`point_size`	Numeric value controlling point size.
`alpha`	Point transparency level (0–1).

`title`	Plot title.
`...`	Additional `ggplot2` layers, scales, or theme adjustments.

Value

A `ggplot` object.

Typical Use

Visualize disease subtypes, clinical risk groups, or molecular classes in PCA space.

`plot_top2_survival_pcs()`

Description

Automatically identifies and plots the two PCs with the smallest adjusted survival p-values.

Usage

```
plot_top2_survival_pcs(
  res,
  meta = NULL,
  color_by = NULL,
  title = "Top 2 Significant PCs",
  ...
)
```

Details

Ensures that displayed PCA structure is directly linked to survival relevance.

2.3 Empirical Null Diagnostics

`plot_null_vs_observed()`

Description

Visualizes the empirical null distribution of a feature's score and overlays the observed score.

Usage

```
plot_null_vs_observed(
  res,
  feature,
  bins = 30,
  hist_fill = "lightblue",
  obs_line_color = "red",
  ...
)
```

Arguments

`feature`	Feature name whose observed score is visualized against the empirical null distribution.
`bins`	Number of bins used in the histogram of null scores.
`hist_fill`	Fill color for the histogram representing the null distribution.
`obs_line_color`	Color of the vertical line indicating the observed feature score.

Value

A `ggplot` object.

Interpretation

- Significant features show strong right-tail separation
- Non-significant features overlap with the null distribution

2.4 Multi-PC Stability Analysis

`run_survival_pca_multi_pc()`

Description

Runs the survival-associated PCA-based scoring pipeline across multiple PC cutoffs to assess feature stability.

Usage

```
run_survival_pca_multi_pc(
  X,
  time,
  status,
  covar = NULL,
  pcs_to_run = c (10, 20, 30, 50),
  ...
)
```

Value

A list containing feature sets, cumulative variance, and settings across PC choices.

`plot_venn()`

Description

Generates a Venn diagram showing overlap of selected feature sets across PC cutoffs.

Usage

```
plot_venn(
  pcset_obj,
  pick,
  label_mode = "both",
  ...
)
```

`plot_feature_set_tradeoff()`

Description

Plots the trade-off between selected feature set size and cumulative variance explained.

Usage

```
plot_feature_set_tradeoff(
  pcobj,
  ...
)
```

Scientific Value

Demonstrates robustness, stability, and interpretability of the selected feature panel as model complexity increases.
