# Supplementary material for: SurvMarker: an R package for identifying survival-associated molecular features using PCA-based weighted scores
Source: BMC Bioinformatics. 2026 May 8;27:167. doi: 10.1186/s12859-026-06461-9 (PMC13449397; doi:10.1186/s12859-026-06461-9)
Supplement: Supplementary file 3 [file 12859_2026_6461_MOESM3_ESM.pdf]

Performance of SurvMarker and competing methods under global null and permutation-based null scenarios.

a

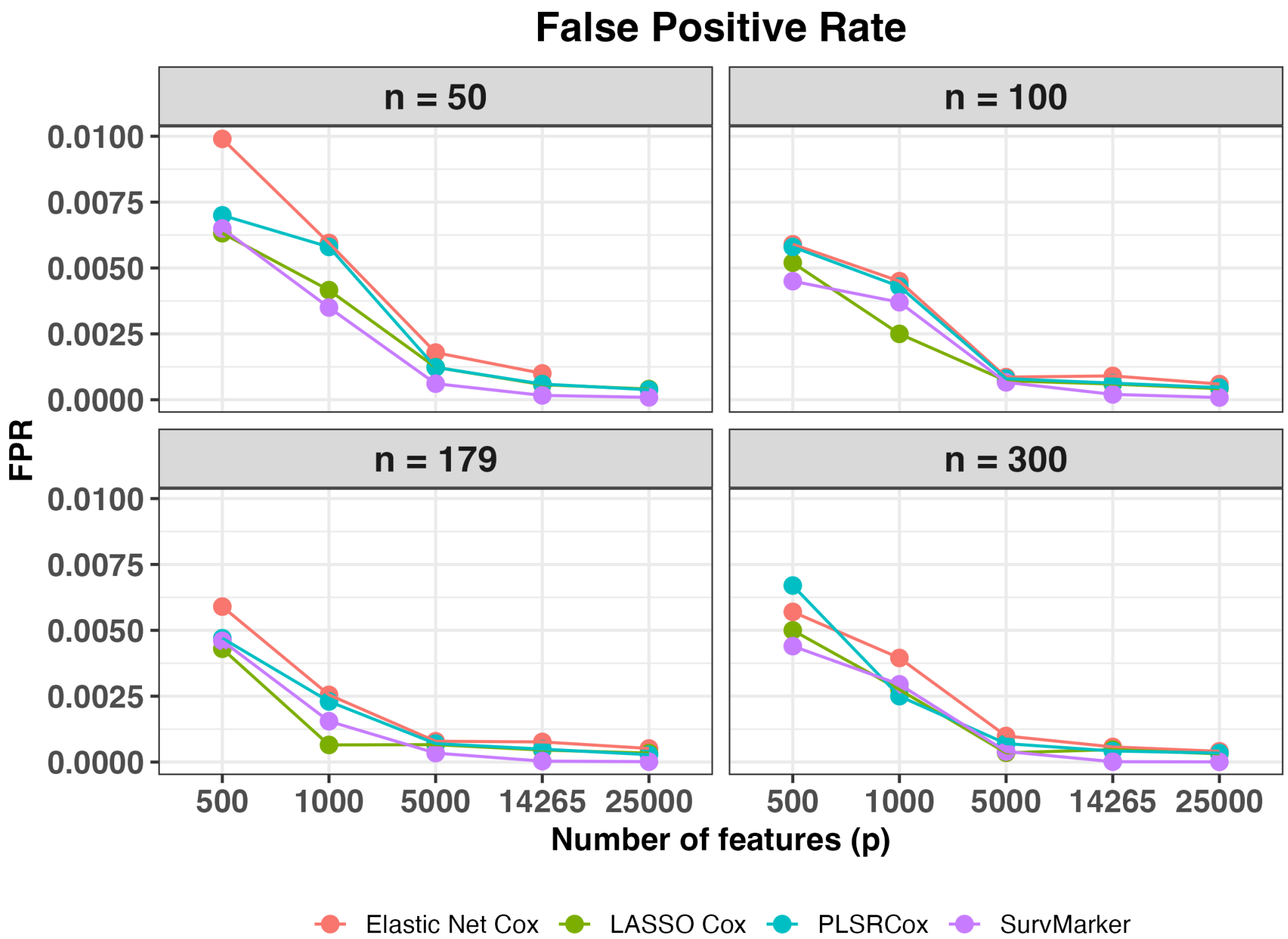

b

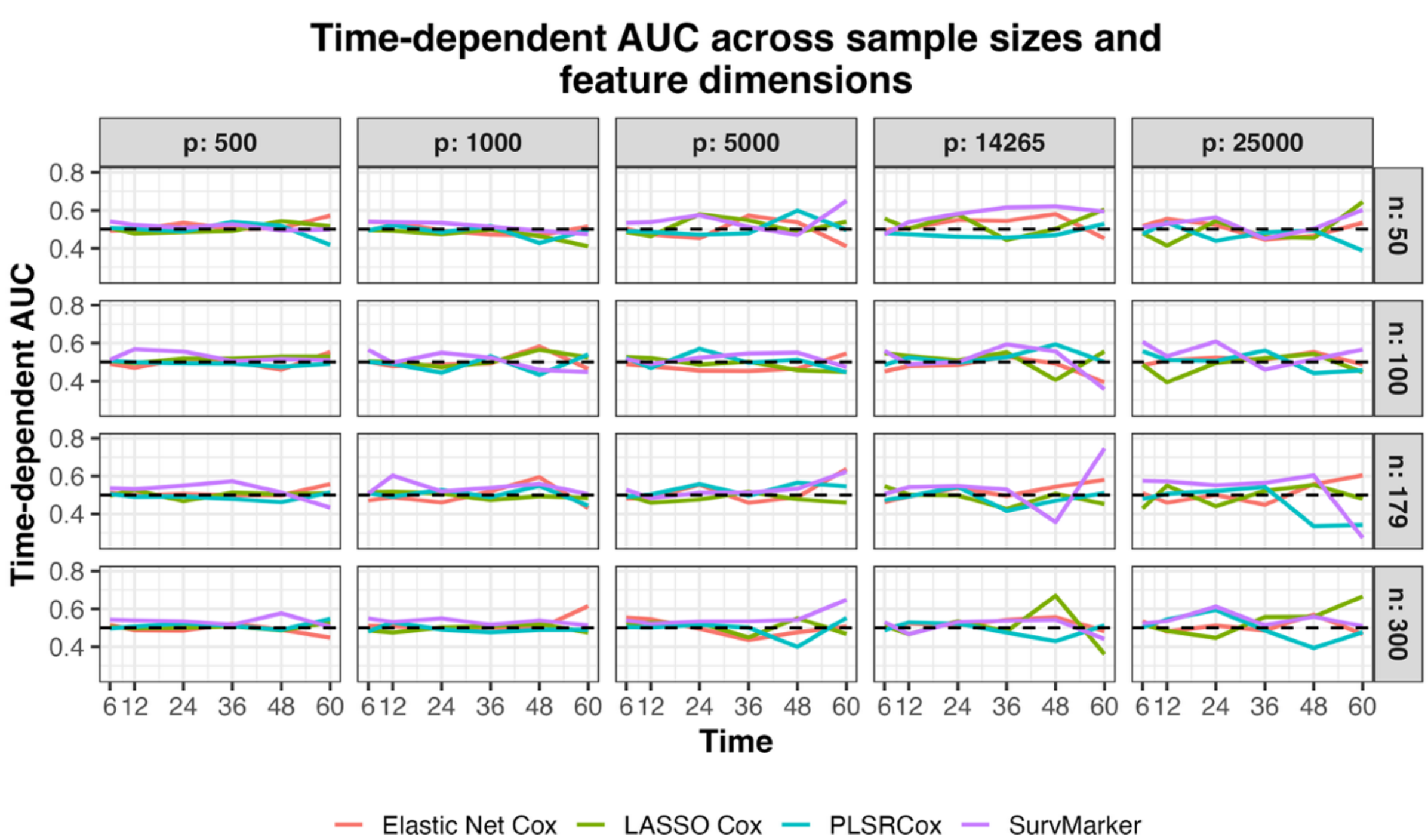

c

	Pairwise Wilcoxon test *p*-values comparing the number of false positives		
	**LASSO Cox**	**Elastic Net Cox**	**PLSRCox**
**SurvMarker**	0.016	0.006	1.28×10^{-8}

d

	Pairwise Wilcoxon test *p*-values comparing the C-index		
	**LASSO Cox**	**Elastic Net Cox**	**PLSRCox**
**SurvMarker**	0.191	0.052	4.46×10^{-4}
