# Supplementary material for: SurvMarker: an R package for identifying survival-associated molecular features using PCA-based weighted scores
Source: BMC Bioinformatics. 2026 May 8;27:167. doi: 10.1186/s12859-026-06461-9 (PMC13449397; doi:10.1186/s12859-026-06461-9)
Supplement: Supplementary file 4 [file 12859_2026_6461_MOESM4_ESM.pdf]

Genes consistently selected across all four methods (LASSO-Cox, Elastic net Cox, PLS-R-Cox, and SurvMarker) representing a stable core signature.

	Gene Symbol
1	DEPDC7
2	MLEC
3	ZNF793
4	CTGF
5	NAGLU
6	MRPL16
7	FHL2
8	MDH2
9	GATA1
10	NCKAP5L
