# Supplementary material for: SurvMarker: an R package for identifying survival-associated molecular features using PCA-based weighted scores
Source: BMC Bioinformatics. 2026 May 8;27:167. doi: 10.1186/s12859-026-06461-9 (PMC13449397; doi:10.1186/s12859-026-06461-9)
Supplement: Supplementary file 5 [file 12859_2026_6461_MOESM5_ESM.pdf]

Comparison of feature selection between variance-based and outcome-driven weighting schemes for the (a) full gene set selected and (b) curated 19-gene prognostic panel.

a

**No. of PCs**	**Outcome-driven (A)**	**Variance-based (B)**	$A \cap B$	**Jaccard Similarity**
10	1355	1355	1355	1.000
20	593	583	556	0.897
30	454	421	411	0.886
50	369	350	339	0.892

b

**No. of PCs**	**Outcome-driven (A)**	**Variance-based (B)**	$A \cap B$	**Jaccard Similarity**
10	16	16	16	1.000
20	19	19	19	1.000
30	15	14	14	0.933
50	19	19	19	1.000
