# Supplementary material for: SurvMarker: an R package for identifying survival-associated molecular features using PCA-based weighted scores
Source: BMC Bioinformatics. 2026 May 8;27:167. doi: 10.1186/s12859-026-06461-9 (PMC13449397; doi:10.1186/s12859-026-06461-9)

Distribution of p-values for principal components not significantly associated with survival.

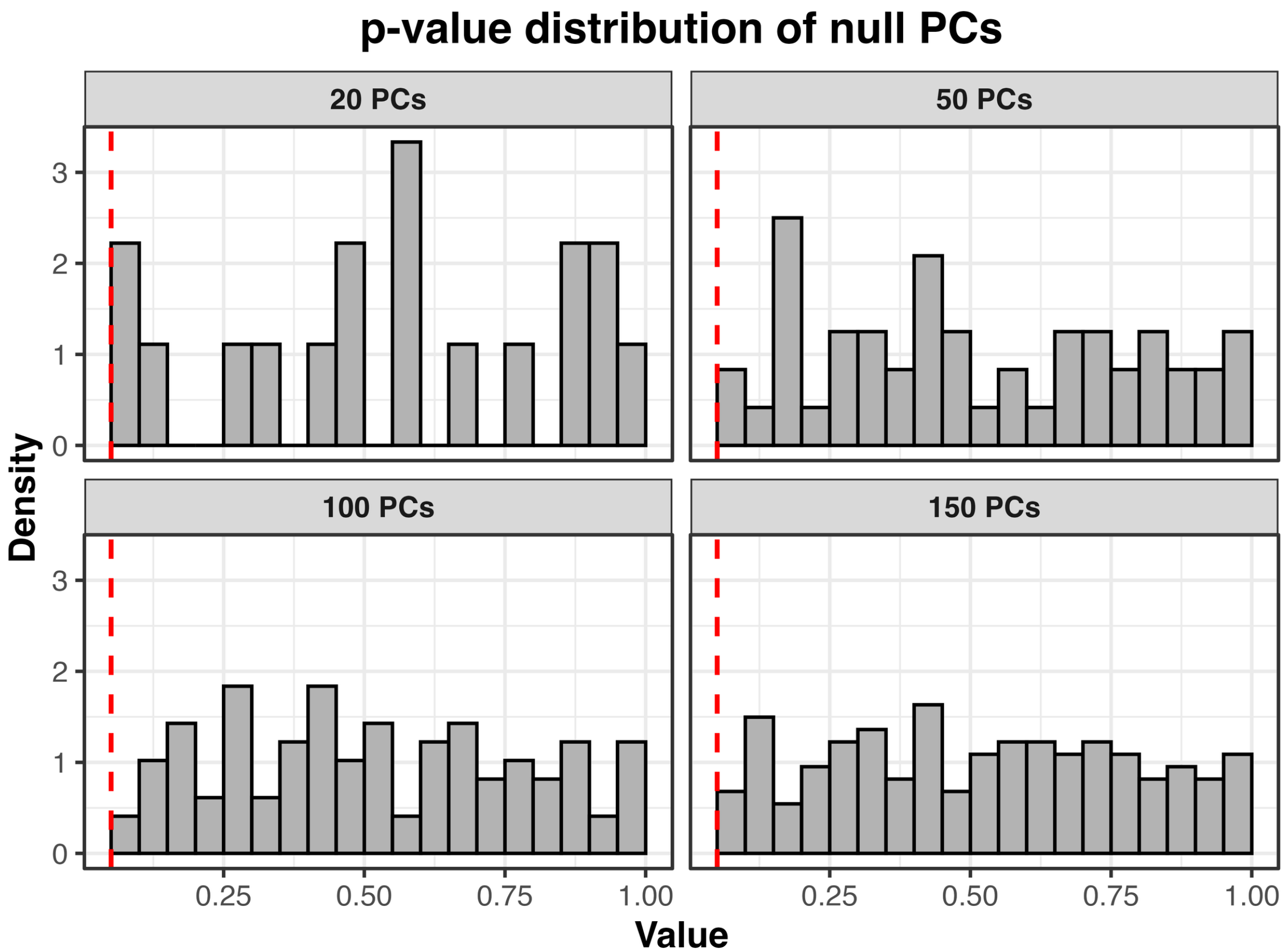

Supplement: Supplementary file 6 [file 12859_2026_6461_MOESM6_ESM.pdf]
