# Supplementary material for: SurvMarker: an R package for identifying survival-associated molecular features using PCA-based weighted scores
Source: BMC Bioinformatics. 2026 May 8;27:167. doi: 10.1186/s12859-026-06461-9 (PMC13449397; doi:10.1186/s12859-026-06461-9)

Performance of SurvMarker and other methods in real data analysis using miRNA expression data from the TCGA-LAML cohort.

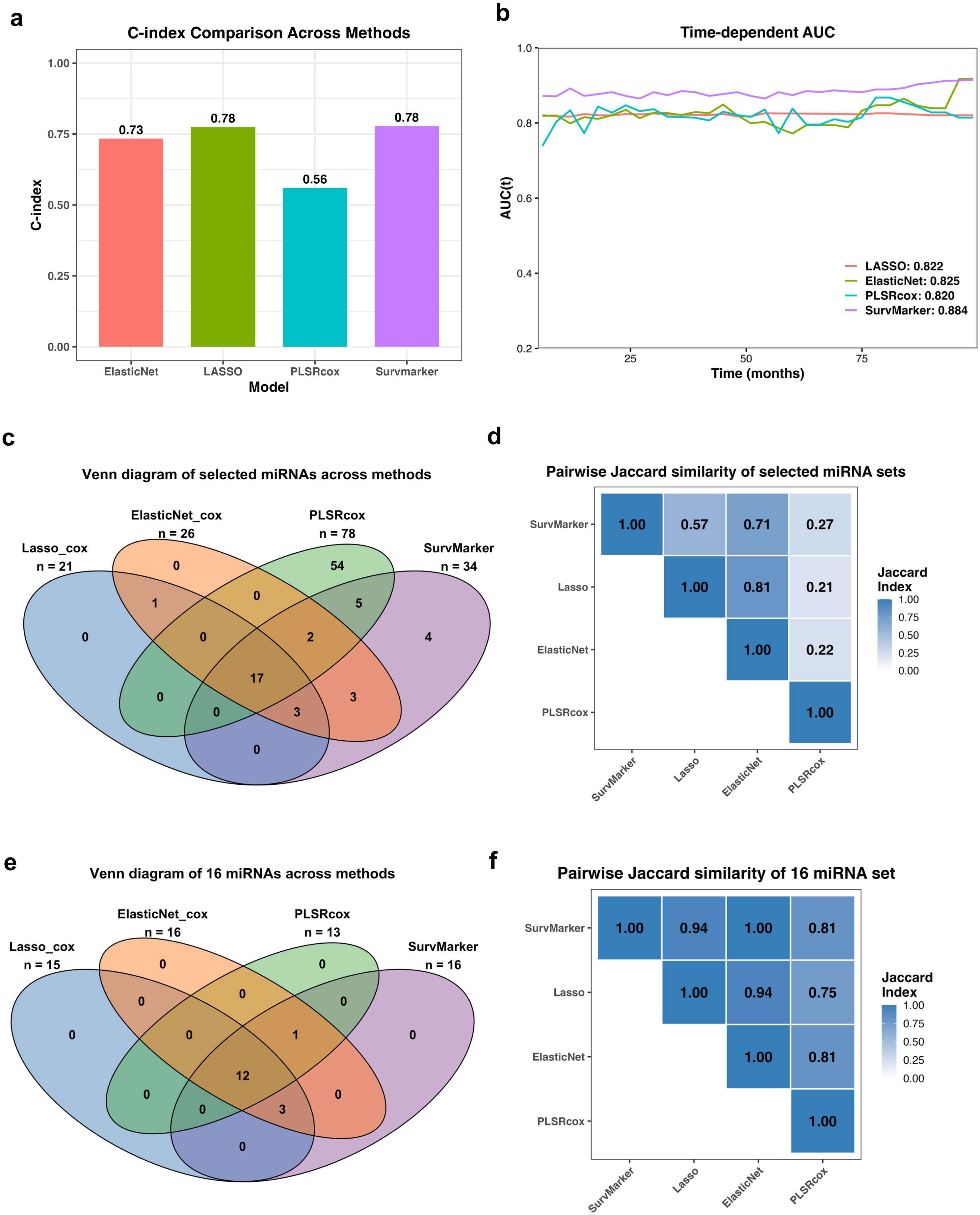

Supplement: Supplementary file 7 [file 12859_2026_6461_MOESM7_ESM.pdf]
